# Highly Efficient Inverted Perovskite Solar Cells with CdSe QDs/LiF Electron Transporting Layer

**DOI:** 10.1186/s11671-017-2381-5

**Published:** 2017-12-06

**Authors:** Furui Tan, Weizhe Xu, Xiaodong Hu, Ping Yu, Weifeng Zhang

**Affiliations:** 1 Key Laboratory of Photovoltaic Materials, Henan University, Kaifeng, 475004 China; 2 Department of Physics and Electronics, Henan University, Kaifeng, 475004 China

**Keywords:** Perovskite, Electron transport, Solar cell, Inverted structure, Quantum dot

## Abstract

Organic/inorganic hybrid perovskite solar cell has emerged as a very promising candidate for the next generation of near-commercial photovoltaic devices. Here in this work, we focus on the inverted perovskite solar cells and have found that remarkable photovoltaic performance could be obtained when using cadmium selenide (CdSe) quantum dots (QDs) as electron transporting layer (ETL) and lithium fluoride (LiF) as the buffer, with respect to the traditionally applied and high-cost [6,6]-phenyl-C61-butyric acid methyl ester (PCBM). The easily processed and low-cost CdSe QDs/LiF double layer could facilitate convenient electron-transfer and collection at the perovskite/cathode interface, promoting an optoelectric conversion efficiency of as high as 15.1%, very close to that with the traditional PCBM ETL. Our work provides another promising choice on the ETL materials for the highly efficient and low-cost perovskite solar cells.

**Electronic supplementary material:**

The online version of this article (doi: 10.1186/s11671-017-2381-5) contains supplementary material, which is available to authorized users.

## Background

Hybrid organic-inorganic perovskite solar cell has been recognized as a very promising new-generation thin film solar cells based on remarkable improvement in its photovoltaic performance with a present efficiency of as high as 22.1% [1]. Long-term environmental stability could also be obtained with a time scale of several hundred to a thousand hours [2, 3]. In the large family of perovskite solar cells, planar heterojunction with an inverted device skeleton has been highly emphasized and intensively researched because of its appealing potential in mild fabrication process and easily accessible flexibility [4–7]. Typically for this device structure, the perovskite layer is sandwiched between the anode and cathode buffer layers to form a p-i-n-layered energy level alignment. In this structure, the n type layer plays a critical role in accepting electrons and inhibiting holes from the perovskite layer.

Up to now, a variety of semiconducting materials were adopted as electron transporting layer (ETL); the traditional choice is the extensively used C_60_ and its derivative, [6,6]-phenyl-C61-butyric acid methyl ester (PCBM) [7–10]. Through uniform and excellent electrical contact with the underlied perovskite film, the small molecule ETLs can provide remarkable efficiency of as high as 19.9% [10]. Although high efficiency was obtained for organic ETLs, gradual attention arises to the high cost of such ETL materials, the complicated device fabrication process, and the unsatisfied device stability. In comparison, ETL materials based on inorganic nanoparticles appeal great attention because of their potential advantage in low material cost, charge mobility, mild fabrication integration, and promising device stability [11–15]. However, up to now, exploring on inorganic ETLs in inverted structure was relatively rare. M. Grätzel and L. Han et al. developed highly conductive Nb-doped TiO2 film on PCBM to obtain an efficiency of 16.2% with > 90% retained PCE after 1000 h of light soaking [12]. Similarly, Alex K et al. introduced Zn2SnO4 nanocrystalline thin film on PCBM buffer layer to facilitate electron extraction and thus increased the device performance to 17.76% [14]. You et al. and Yang et al. firstly fabricated all-metal-oxide layer-based inverted perovskite solar cell that show 16.1% efficiency and significantly improved stability [15]. Generally, either the quantity of reported works or the photovoltaic performance of this inverted devices lagged behind the traditional structure. More investigation on the inorganic ETL-based inverted perovskite solar cells are needed to accelerate the fast growth of this field.

Here in this work, we developed a novel all-inorganic ETL for the inverted perovskite solar cells, a cadmium selenide (CdSe) quantum dots (QDs)/lithium fluoride (LiF) double layer obtained from spin-coating and thereafter evaporation process. Up to now, the synthesis and optoelectric application of CdSe QDs have been extensively reported as electron acceptor [16–18]. Ultrathin and island-shaped LiF were also widely used in the cathode buffer layers in organic solar cells [19, 20]. All these well-developed references prompt us to consider them as inorganic ETL and cathode buffer layer in the inverted perovskite solar cells. We have found that the CdSe/LiF layer plays an excellent role in extracting and transferring electrons from the underlying perovskite to the above cathode, enabling a photovoltaic conversion efficiency of as high as 15.1% that is very close to the PCBM reference. Our work provides another promising choice on the low cost and all-inorganic electron extraction layer for inverted perovskite solar cells.

## Methods

### Synthesis of CdSe QDs

Cadmium oxide (CdO, 1 mmol), oleic acid (OA, 10 mmol), and 3 g trioctylphosphine oxide (TOPO) were dissolved in a four-neck round bottom flask and pumped at 140 °C under N_2_ flow for 30 min. After that, the temperature was raised to about 280 °C during which the solution turned clear. A TOP-Se solution (containing 1 mmol Se in 3 ml tri-n-octylphosphine (TOP) was injected into the flask quickly. The reaction was allowed at 260 °C for 4 min, and then, the heating mantle was removed. After the solution was cooled to room temperature, 10 ml acetone was injected to collect the red precipitation by centrifugation at 4500 rpm. The obtained CdSe QDs were cleaned with chlorobenzene (CB)/acetone solvent/antisolvent for at least four times and then dissolved in 30 ml pyridine and stirred at 50 °C overnight to exchange the surface OA ligands. Then, the pyridine-capped CdSe QDs were collected by adding n-hexane to the solution and thereafter centrifuging at 4000 rpm. About 8 ml CB was used to disperse the collected CdSe QDs. The concentration of the final solution was adjusted to 15 mg/ml that was used for solar cell fabrication.

### Device Fabrication

The pre-patterned indium tin oxide (ITO) glass was firstly untrasonicated with deionized water, acetone, and isopropanol separately for 30 min and then dried by N_2_ blowing. One hundred microliter poly(3,4-ethylenedioxythiophene) poly(styrene-sulfonate) (PEDOT:PSS, VPAI 4083) was spin-coated onto the ITO at 6000 rpm and then dried at 120 °C in air. The organic-inorganic perovskite solution was prepared by mixing 2 mmol MAI and 2 mmol PbI_2_ in 1.6 ml DMF. The solution was stirred at 70 °C overnight in N_2_-filled glovebox. The perovskite film was deposited on the substrate through a two-step spin-coating procedure (1000 rpm for 10 s and 6000 rpm for 30 s). One hundred eighty microliter chlorobenzene was deposited quickly at 5 s since the beginning of the second stage of spin-coating. All the perovskite films were annealed at 100 °C for 10 min. After cooling down, the as-prepared CdSe QD chlorobenzene solution was dripped on the perovskite surface, stayed for 5 s, and then spin-coated at different speed to obtain different film thickness. The substrate was transferred into thermal evaporator where a 0.8–1.0-nm LiF ultrathin film or particle islands was deposited (0.2 Å/s, 6 × 10^−4^ Pa) followed by 20 nm Au and 80 nm Ag. A mask was used to define six separate pixels each with an effective area of 0.04 cm^2^.

### Measurements

The film topology with and without CdSe/LiF covering were researched by field emission scanning electron microscope (FESEM, JEOL 7006F) and scanning probe microscope (SPA400). X-ray diffraction (XRD) was performed on a Rigaku D/max-gA X-ray diffractometer with Cu Kα radiation. Light absorption properties were measured with ultraviolet-visible-inferred spectrophotometer (Varian Cary-5000). Photoluminescence (PL) spectra were collected on HORIBA Jobin Yvon Fluorlog-3 system. Time-resolved photoluminescence (TRPL) spectroscopy measurements were conducted using a pulse laser (512 nm) for excitation (F980 lifetime spectrometers, Edinburgh Instruments, EI). The TRPL decays at 790 nm were recorded by a time-correlated single-photon counting (TCSPC) spectrometer. The photovoltaic *I*-*V* properties were recorded on Keithley 2440 source meter combined with Newport 94043A solar simulator (AM 1.5 illumination). The unencapsulated solar cells were tested at room temperature in air. Typically, light soaking was needed to get a stabilize power conversion efficiency. External quantum efficiency (EQE) was measured on a solar cell IPCE measurement system (Crowntech Qtest Station 500ADX) with a CM110 monochromator, a Keithley 2000 source meter, and a CT-TH-150 Br-W lamp. The surface photovoltage (SPV) spectrum were obtained from a measurement system containing the source of monochromatic light, a lock-in amplifier (SR830-DSP) with a light chopper (SR540). Electrochemical impedance spectra (EIS) were measured from a CHI 660E electrochemical workstation (Chenhua Inc., Shanghai), applying a 10-mV AC signal and scanning in a frequency range between 1 MHz and 1000 Hz at different forward applied bias.

## Results and Discussion

**Fig. 1:**
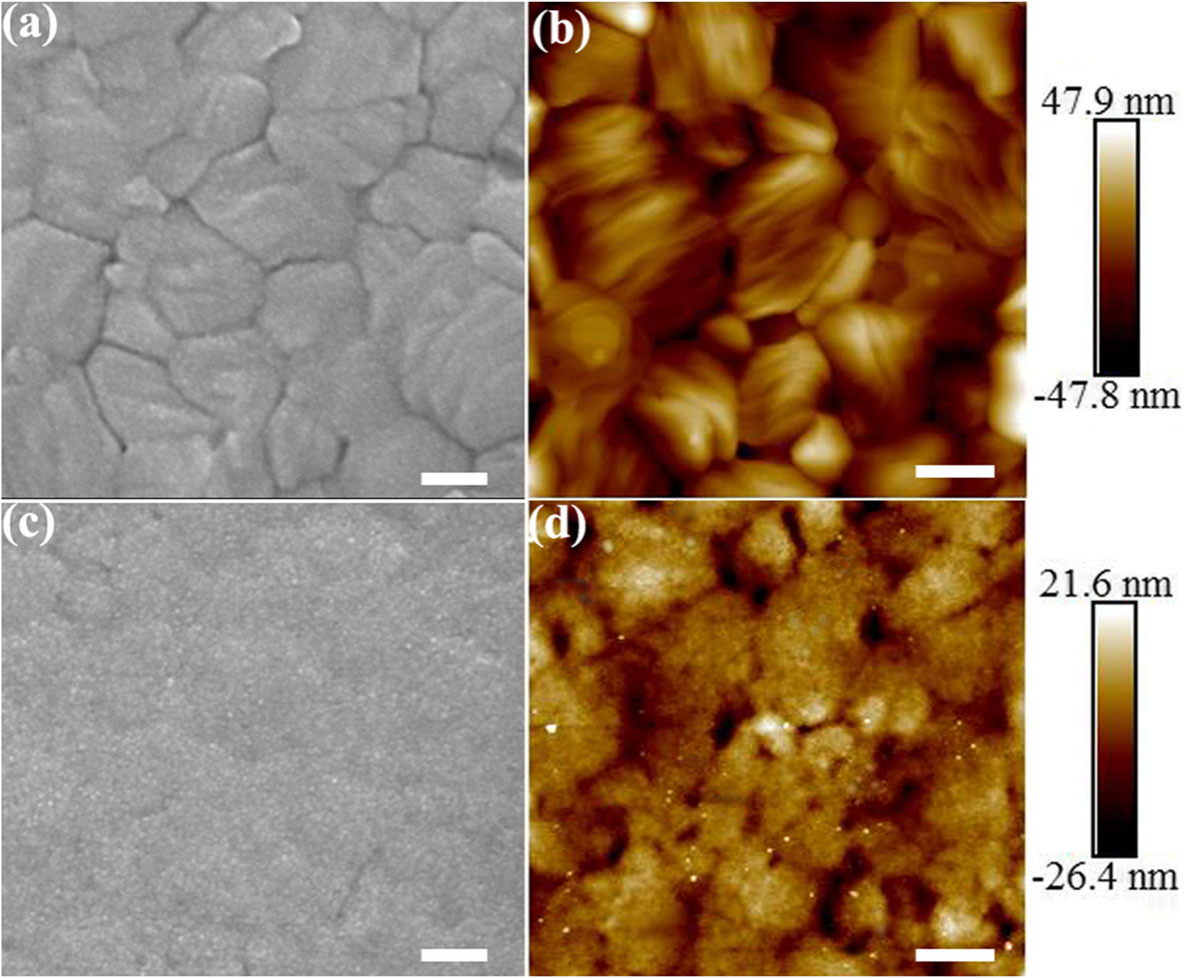
SEM and AFM topology of bare perovskite film (**a**, **b**) and CdSe/LiF-covered perovskite film (**c**, **d**)

The MAPbI3-based perovskite films were fabricated with the traditional one-step process with chlorobenzene as the antisolvent. The bare perovskite film shows a very flat surface without any large pinholes and cracks (Fig. 1a). AFM test further confirms the densely packing of perovskite crystals mostly with a size of about 500–700 nm (Fig. 1b). Crystal boundaries can be clearly observed from both the SEM and AFM images. After deposition of CdSe/LiF, the surface seems sandy and flatter, indicating that the perovskite crystals as well as their boundaries are readily covered with tiny CdSe QDs and LiF (Fig. 1c). This is also reflected from the corresponding AFM image (Fig. 1d). Some hazy boundary outlines are still observable from the SEM and AFM images, indicating that the covered CdSe/LiF double layer has a very small performance-optimized thickness. As the wurtzite phase CdSe QD has an averaged diameter of about 5.5 nm (Additional file 1: Fig. S1) and the modified LiF layer is just 0.8–1.0 nm, exact distinction of the two materials is difficult. The root mean roughness (RMS) of the film surface decreases from 10.6 nm for the bare perovskite to 4.7 nm for the CdSe/LiF deposited. Thus, fully contacted perovskite/ETL interface provides spatial convenience for electron transfer and collection through the above CdSe/LiF double layer.

**Fig. 2:**
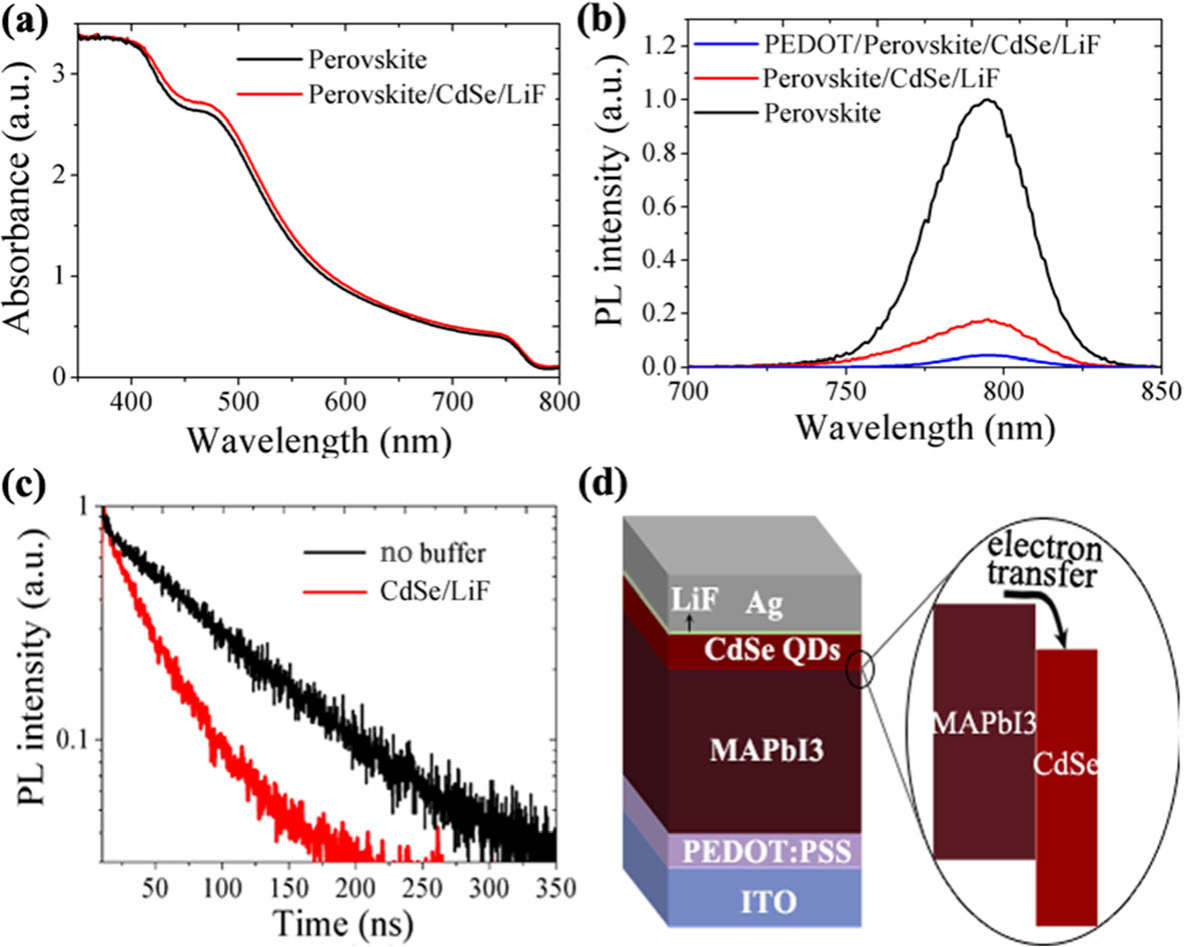
Light absorption (**a**), photoluminescence (**b**), and time-resolved PL spectrum of the perovskite films with and without ETL layer (**c**). Device skeleton and energy level alignment at the interface (**d**)

The absorption properties of the films with and without CdSe/LiF layer are shown in Fig. 2a. The bare MAPbI3 film shows a strong absorption at the entire visible region, with a typical absorption startup at about 770 nm. After depositing the CdSe/LiF on top, the film shows similar absorption tendency without much variation. Slightly increased absorption intensity in the visible light region is probably ascribed to more light scattering from the top QD layer. As the thickness of CdSe QD layer is much thinner than that of the perovskite film, the characteristic absorption of CdSe QDs (Additional file 1: Fig. S2) is not clearly exhibited.

To evaluate the charge transfer and collection ability of this novel perovskite/CdSe interface, we characterized the photoluminescence (PL) properties of different samples. The bare MAPbI_3_ film on ITO glass shows a strong PL peak at about 790 nm (Fig. 2b) while this peak intensity is up to 80% quenched for the sample covered with CdSe/LiF layer. This result reflects that the photon-generated charges could be effectively separated at the perovskite/CdSe interface. Incorporation of the PEDOT:PSS anode buffer layer beneath the perovskite layer further quenches the PL intensity. For further evidence, time-resolved photoluminescence (TRPL) decay spectrum were characterized to probe the effect of inorganic buffer layer on carrier dynamics in the solar cells. For pure perovskite film, it was reported that a longer PL lifetime could be obtained through suppressing charge recombination with mixed antisolvent or surface passivation [21, 22]. Here in this work, we focused on chlorobenzene for easy comparison, although other antisolvent may also play a positive role in fabrication of uniform perovskite films [23]. The results in Fig. 2c show that the TRPL signal of perovskite film covered with CdSe/LiF has a faster decay as compared to the film without cathode buffer, indicating a rapid charge injection from MAPbI3 to CdSe. As shown in Fig. 2d, the perovskite/CdSe contact could form a typical type-II heterojunction that facilitates exciton dissociation and charge transfer. Thus, the results demonstrate that the adopted CdSe QDs/LiF layer is electronically beneficial to charge extraction as a cathode buffer layer. Therefore, it is highly expectable to gain a reasonable photovoltaic performance by applying the PEDOT:PSS/MAPbI3/CdSe/LiF heterostructure. The planar solar cell was thus fabricated with CdSe QDs and PEDOT:PSS as the cathode and anode buffer layer respectively, as is shown in Fig. 2d.

**Fig. 3:**
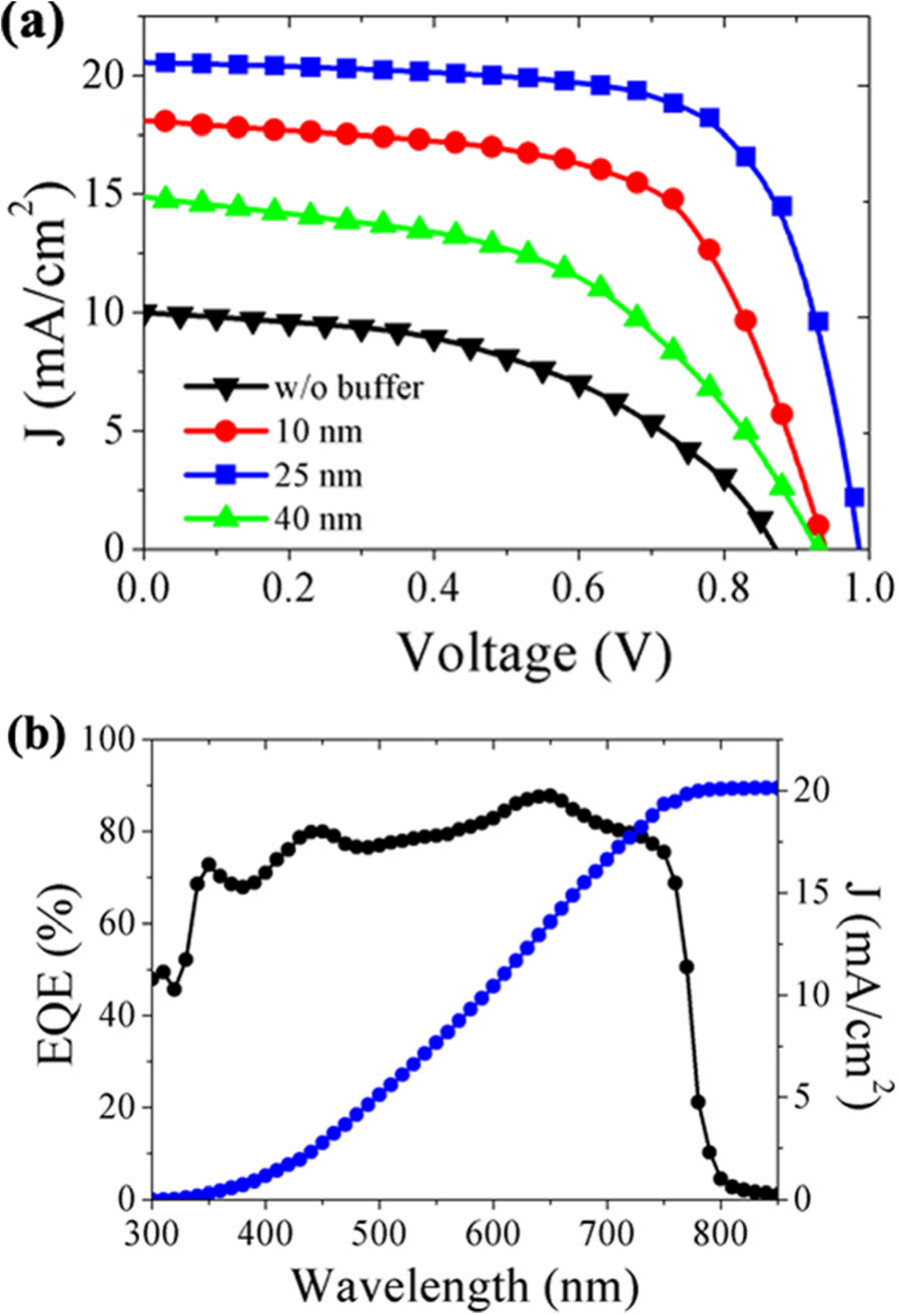
Photovoltaic performance of solar cells w/o and with CdSe QD layers with different thickness (**a**). External quantum efficiency and the integrated current density of the optimized solar cell (**b**)

The photovoltaic performance of solar cell without ETL was also fabricated and measured as reference. The performance stability and repeatability of this device were found to be very poor. The best device obtained in our work generated a voltage-oriented control (Voc) of 0.88 V, current density (Jsc) of 10 mA/cm^2^, fill factor (FF) of 48%, and a conversion efficiency of 4.2% (Fig. 3a). Introduction of CdSe/LiF buffer layer could remarkably enhance the performance. A 10-nm CdSe QDs layer could generate greatly enhanced performance while a double layer containing 25 nm CdSe and 1 nm LiF atop produces the best target solar cells. An averaged conversion efficiency of 14.2% is achieved with a Voc of 0.99 V, a Jsc of 20.5 mA/cm^2^, and an FF of 69.9%. Further increase of the CdSe QDs layer thickness will deteriorate the performance due to largely increased series resistance (Table 1). It is noticed that this performance could only be obtained with CdSe QDs capped with pyridine. The original OA ligand always plays a detrimental role on charge transfer and collection, exhibiting an S-shaped *I*-*V* curve (Additional file 1: Fig. S3). The excellent photovoltaic performance from adopting CdSe/LiF buffer layer is also confirmed by the EQE results (Fig. 3b). Integration of the EQE values generates a Jsc value of 20.2 mA/cm^2^ that is very close to the above measured one. It is noticed that performance obtained with our modified buffer layer is among the top values of reported perovskite solar cells with some other buffer layers [14, 15], showing the promising effectiveness of this novel ETL.

**Table 1:** Photovoltaic performance of inverted MAPbI3 solar cells with different thickness (*T.*) of CdSe QD layer

*T.* (nm)	Voc (V)	Jsc (mA cm^−2^)	FF (%)	Eff (%)	Rss (Ω)
0	0.88	10.0	47.7	4.2	29
10	0.94	18.1	63.5	10.8	16
25	0.99	20.5	69.9	14.2	9
40	0.93	14.9	50.0	6.9	31

**Fig. 4:**
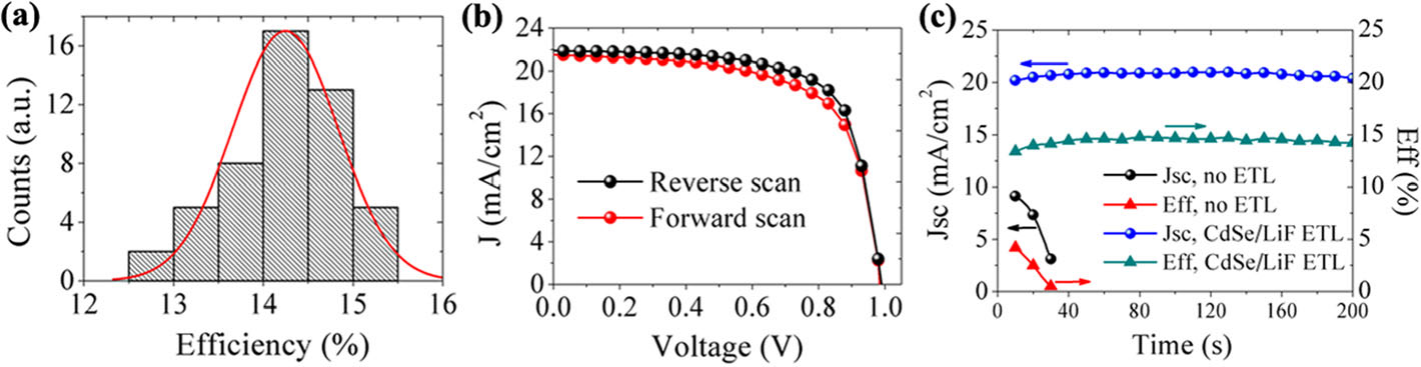
Performance statistics of the solar cells (**a**), *I*-*V* curves of the forward and reverse-can mode of the best solar cell (**b**) and comparison of performance stability of the solar cells with and without ETL (**c**)

To further confirm the adaptability of the CdSe QDs/LiF layer, the performance data from over 50 devices in different batches were collected. Figure 4a shows the efficiency statistics of the obtained solar cells. The efficiency distribution is a little large with an average value of 14.2%; the best and the worst devices generate an efficiency of 15.1 and 12.7%, respectively. Normally, we synthesized fresh CdSe QDs for every batch of solar cells’ fabrication. The QD quality may cause performance fluctuation among different batches because of the occasional aggregation of QDs during ligand exchange. However, near the averaged value, the solar cells exhibit a good repeatability. The best device shows no appreciable hysteresis during the reverse and forward scans (Fig. 4b). Besides, we notice this efficiency maximum of the CdSe/LiF ETL device is close to that of a traditional PCBM ETL with a maximum efficiency of 16.14% (Additional file 1: Fig. S4). For device stability, we tracked its performance under continuous light illumination. The solar cells with CdSe/LiF show a little increase in performance at the beginning of illumination due to light-soaking effect that was commonly observed in perovskite solar cells [24, 25]. It should be noted that the *I*-*V* measurement was started after the initial explosion to light for about 5 s. So the performance stability was recorded after 5 s from light illumination (Fig. 4c). It can be seen that the current density as well as the conversion efficiency is stable during the light-soaking time shown, meaning that the perovskite solar cells with CdSe/LiF ETL are stable. However, without ETL covering, the solar cells show a drastical decrease during the first several seconds of illumination. This result demonstrates that our buffer layer could readily play a positive role in inhibiting moisture and oxygen that could cause fast deterioration of solar cells’ performance.

**Fig. 5:**
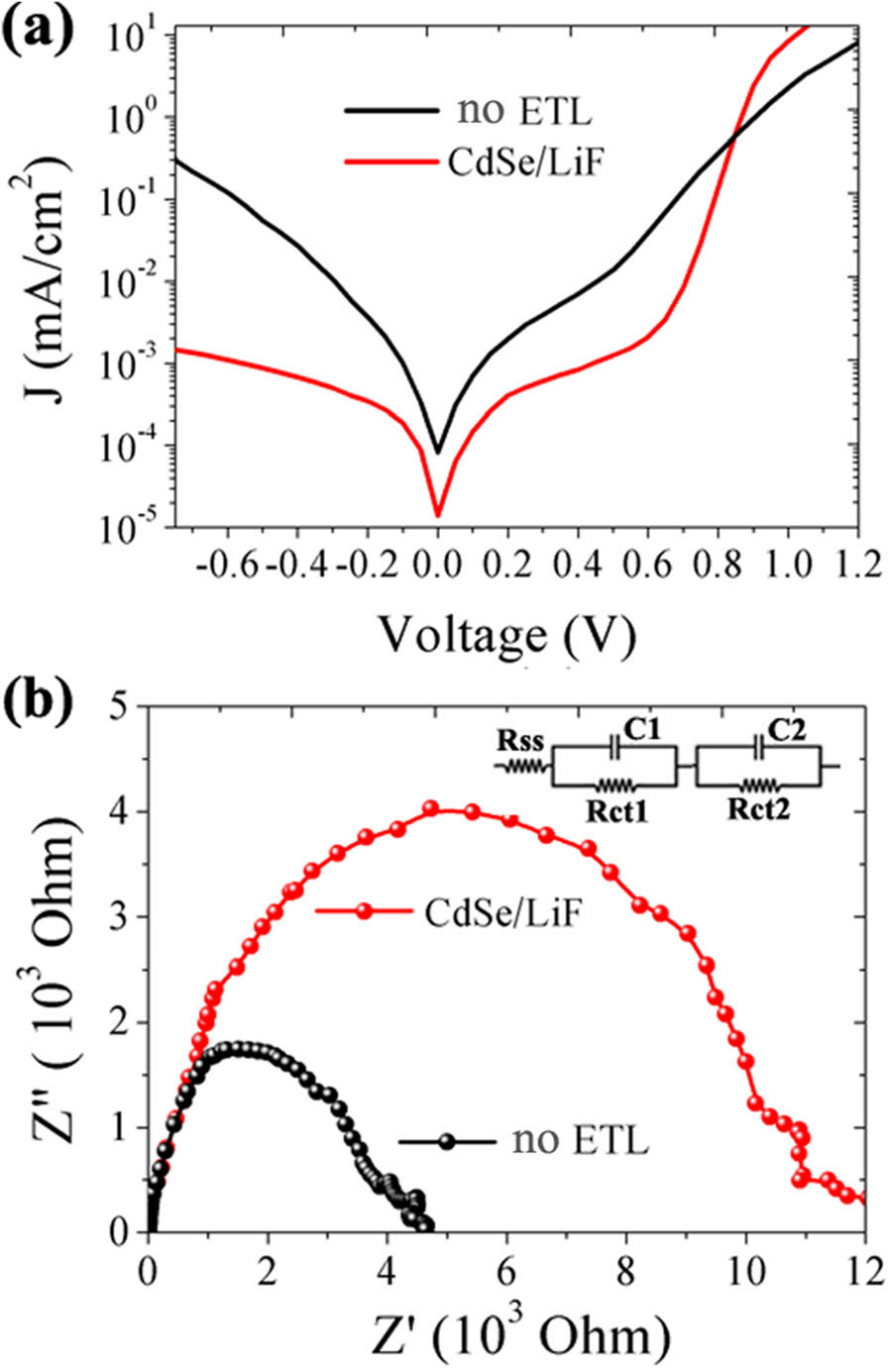
Dark current density (**a**) and electrochemical impedance spectrum (**b**) of the solar cells with and without ETL

As the electron extraction layer, the CdSe/LiF should efficiently collect electrons and inhibit holes from the perovskite film. Figure 5a shows the dark current density at different bias voltage. The reference device shows large current leakage due to the absence of cathode buffer layer. On the other hand, a much better rectify factor was obtained by introducing CdSe/LiF ETL and therefore, the current leakage is reduced. Further characterization on this property is carried out through electrochemistry impedance spectrum (EIS). Figure 5b shows the EIS results of the two devices under dark in an open-circuit condition. Compared to the reference, the target device shows a larger diameter of the semicircle, that is, a larger charge recombination resistance in the perovskite film and at the perovskite/ETL interface [26, 27]. The addition of perovskite/CdSe interface could increase charge transfer recombination resistance (Rct) value as shown in inset of Fig. 5b, which indicates a decreased charge recombination near the cathode. Thus, our results demonstrate an enhanced charge transfer and extraction through the CdSe/LiF ETL.

**Fig. 6:**
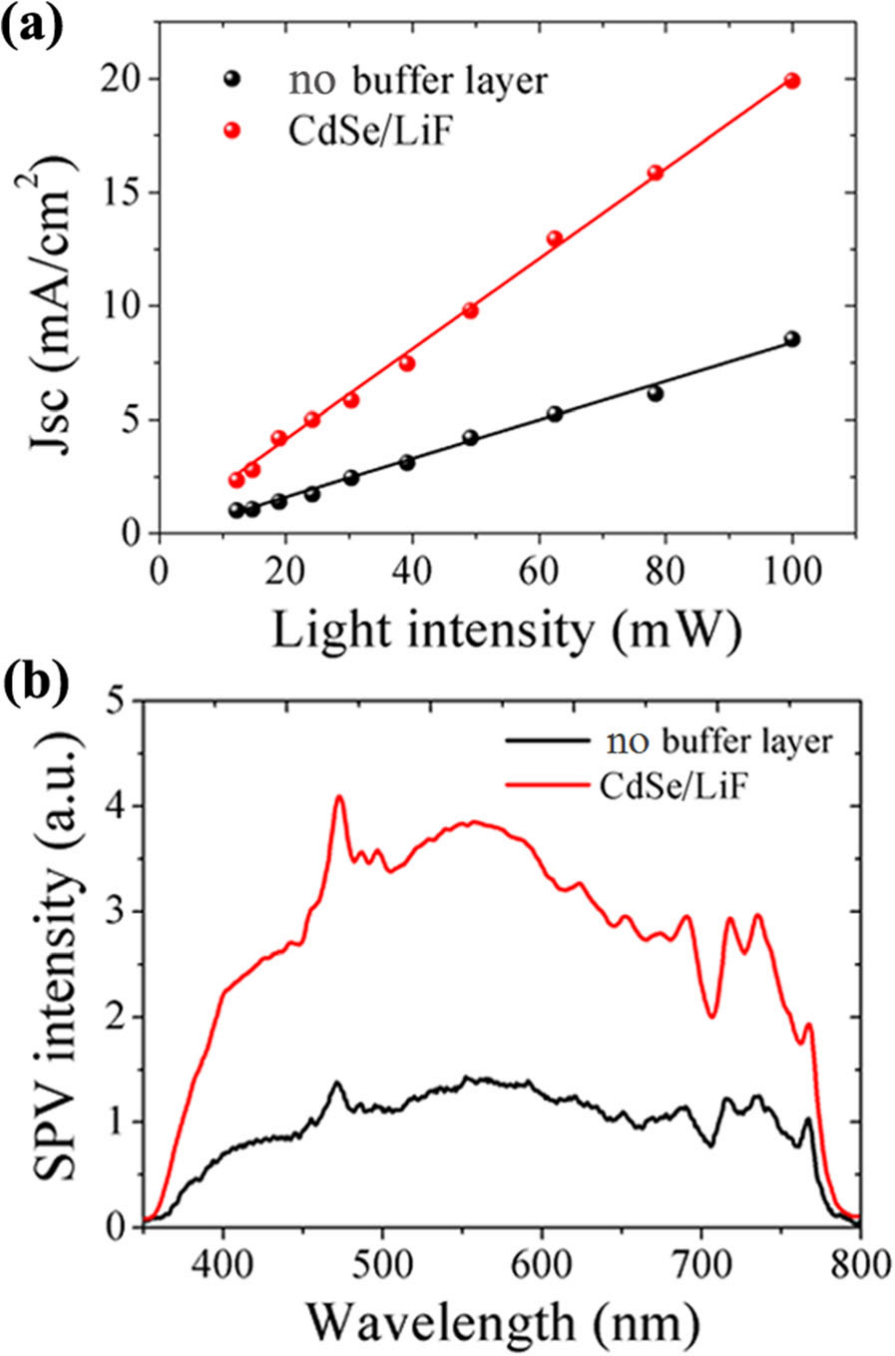
Light intensity dependence of current density (**a**) and surface photovoltage spectrum (**b**) of the solar cells

To further evaluate the charge collection ability of this buffer layer, we characterized the short-circuit current density under different light intensity and the results are shown in Fig. 6a. Both of the two devices show a near-linear increase in Jsc following the increase in light intensity. The CdSe/LiF device exhibits a much faster increase than the reference, demonstrating enhanced charge collection ability under higher light intensity. This property is also indicated from the surface photovoltage spectrum (SPV) in Fig. 6b. Without a buffer layer, the device generates relatively weak SPV signals in the visible light region, while the adoption of CdSe/LiF layer greatly enhances the SPV values in the same region. As the SPV signal is correlated with charge generation and thereafter transportation to the film surface [17, 28], the larger SPV value in the target device could be reasonably explained by the enhanced charge collection and transportation through a type-II heterojunction at the perovskite/ETL interface, as is shown in Fig. 2d.

## Conclusions

In conclusion, we have fabricated planar perovskite solar cells with CdSe quantum dots/LiF electron transporting layer that is compatible to the solution process of the device. The uniform and full coverage of perovskite film through a 25-nm CdSe QDs and 1 nm LiF would provide spacial and electronic convenience for electrons’ transfer and extraction, as indicated from the TRPL, EIS, and SPV characterization and so on. The adoption of this ETL brings a significant increase in photovoltaic efficiency, from 4.8% for that without buffer layer to 14.2% in the optimized target and a maximum of 15.1%. The performance stability is also improved. Our work provides a promising candidate on ETLs for the development of highly efficient and low-cost inverted perovskite solar cells.

## Additional file

Additional file 1: Figure S1. (**a**) TEM image of synthesized CdSe quantum dots (QDs), (**b**) QDs size statistics and (**c**) XRD pattern of synthesized CdSe QDs. The result shows a wurtzite phase of synthesized material. **Figure S2.** Light absorption of the synthesized CdSe QDs chlorobenzene solution. **Figure S3.** *I*-*V* performance of solar cell with OA capped CdSe QDs. **Figure S4.** The best photovoltaic performance of perovskite solar cells with traditional PCBM as ETL.
