# Supplementary material for: Highly Efficient Inverted Perovskite Solar Cells with CdSe QDs/LiF Electron Transporting Layer
Source: Nanoscale Res Lett. 2017 Dec 6;12:614. doi: 10.1186/s11671-017-2381-5 (PMC5718992; doi:10.1186/s11671-017-2381-5)
Supplement: Additional file 1: Figure S1. — (a) TEM image of synthesized CdSe quantum dots (QDs), (b) QDs size statistics and (c) XRD pattern of synthesized CdSe QDs. The result shows a wurtzite phase of synthesized material. Figure S2. Light absorption of the synthesized CdSe QDs chlorobenzene solution. Figure S3. I-V performance of solar cell with OA capped CdSe QDs. Figure S4. The best photovoltaic performance of perovskite solar cells with traditional PCBM as ETL. [file 11671_2017_2381_MOESM1_ESM.doc]

**Supporting Information**

**Highly efficient inverted perovskite solar cells with CdSe QDs/LiF electron transporting layer**

Furui Tan^1,2^, Weizhe Xu^1,2^, Xiaodong Hu^1,2^, Ping Yu^1,2^, Ling Wei^1,2^, Weifeng Zhang^1,2^.

*^1^ Key Laboratory of Photovoltaic Materials, Henan University, Kaifeng, 475004, China.*

*^2^ Department of Physics and Electronics, Henan University, Kaifeng, 475004, China.*


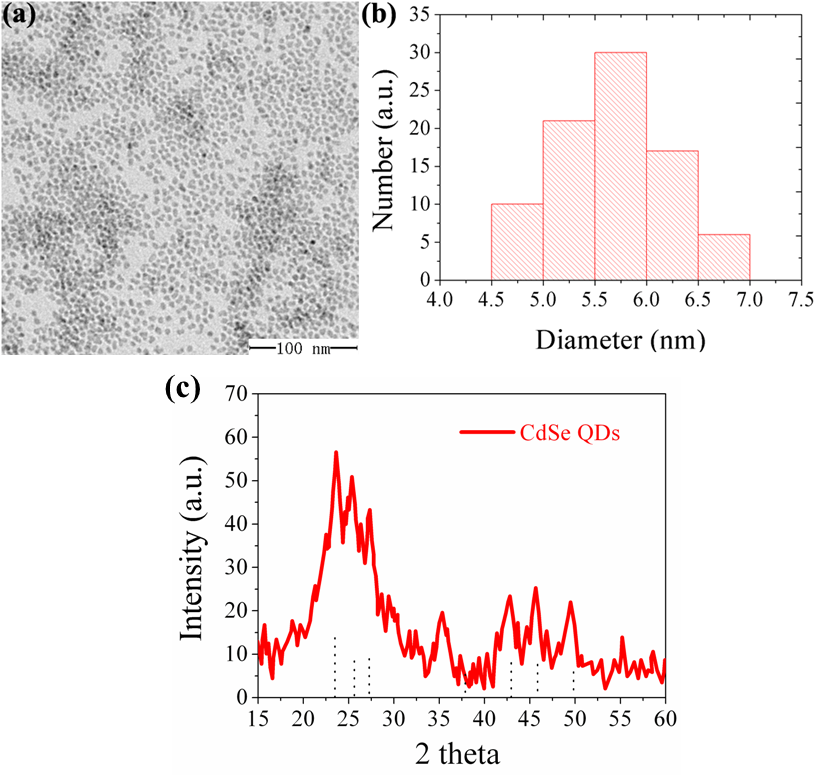


Fig. SI1. (a) TEM image of synthesized CdSe quantum dots (QDs), (b) QDs size statistics and (c) XRD pattern of synthesized CdSe QDs. The result shows a wurtzite phase of synthesized material.


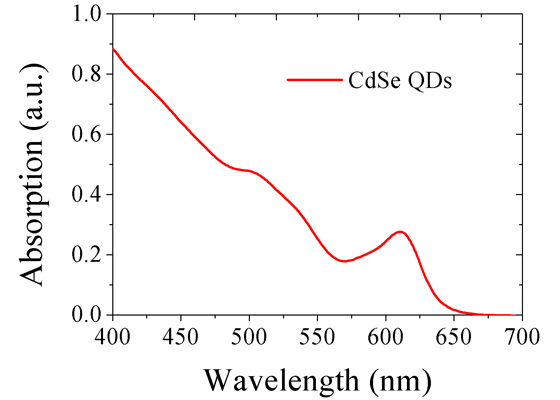


Fig. SI2. Light absorption of the synthesized CdSe QDs chlorobenzene solution.


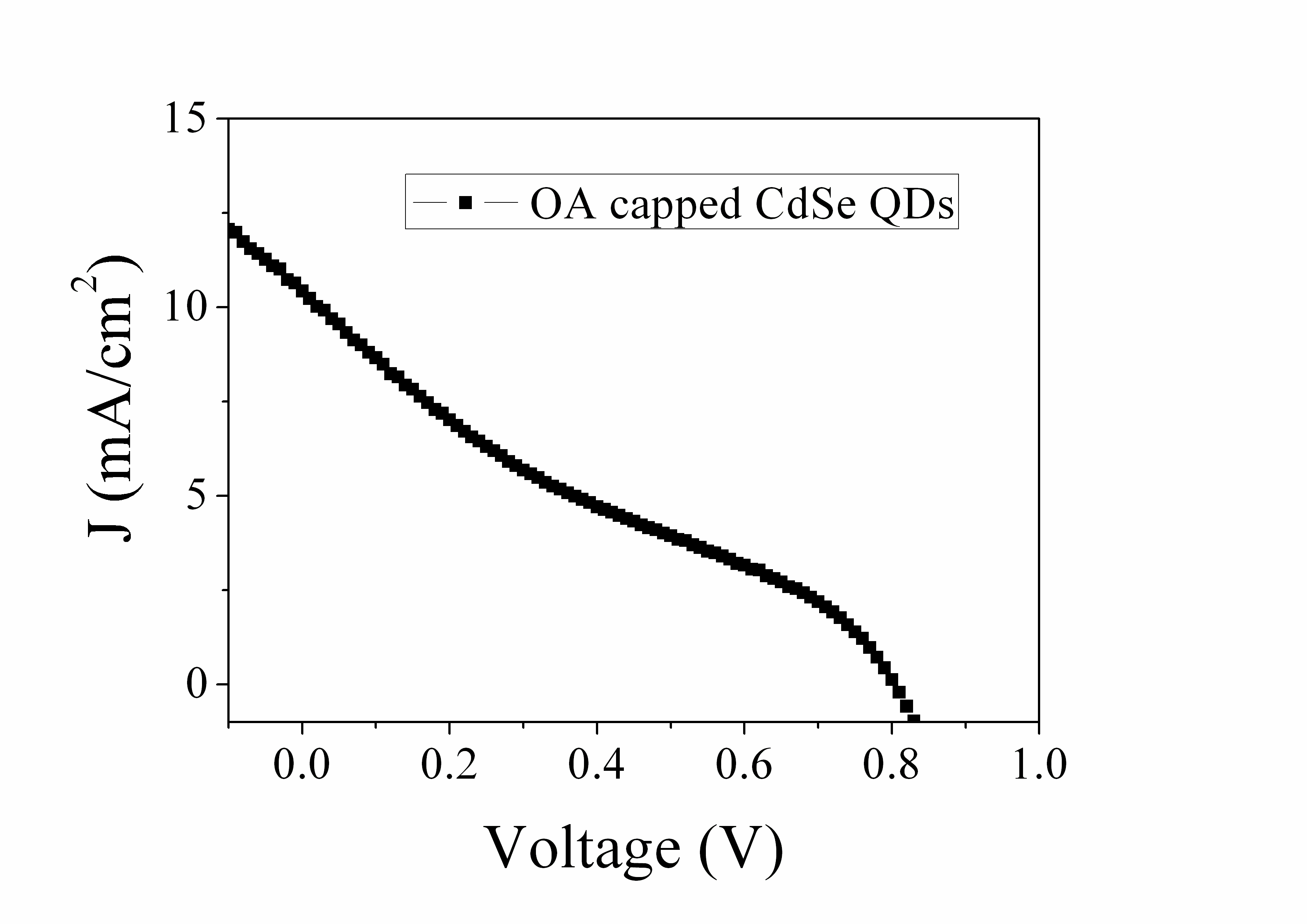


Fig. SI3. IV performance of solar cell with OA capped CdSe QDs.


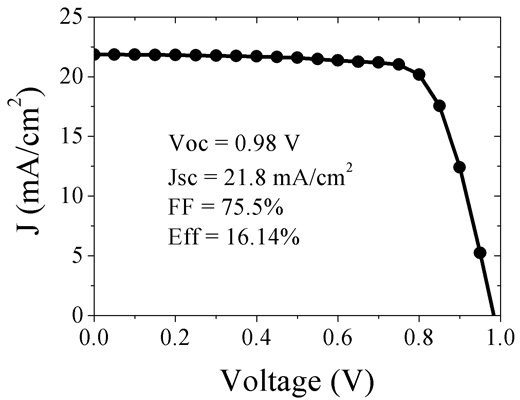


Fig. SI4. the best photovoltaic performance of perovskite solar cells with traditional PCBM as ETL.
